# Supplementary material for: Patterns of Intron Gain and Loss in Fungi
Source: PLoS Biol. 2004 Nov 30;2(12):e422. doi: 10.1371/journal.pbio.0020422 (PMC532390; doi:10.1371/journal.pbio.0020422)
Supplement: Table S1 — Also available at http://genes.mit.edu/NielsenEtAl/. (4.3 MB ZIP). [file pbio.0020422.st001.zip › NielsenEtAl/html/1324.html]

AN6557.1.NCU00418.1.MG06310.1.FG06936.1


```
 CLUSTAL W (1.82) Multiple Sequence Alignments - Introns Inserted


Sequence 1: MG06310.1	124 aa
Sequence 2: FG06936.1	124 aa
Sequence 3: NCU00418.1	131 aa
Sequence 4: AN6557.1	123 aa
Alignment Length: 131 aa
Number Identitical Residues: 62 aa
Alignment Score (without introns) 3300


MG06310.1 	MAITPTEFAKRTAQS1ANWADAKRRVLSSYREWLRA0APEIQTMYSVTHPVSTIRTRIRQ
NCU00418.1	MPITPTKYAITTRQS1ANWSDAKRRVFALYRRWLRS0TPEMQSMYSLPLPISVIRTRIRQ
FG06936.1 	MAITPTQFAKKTAQS1ANWSDAKRRVLSSYREWIRA0APEVQTMYNMPMPVSTIRTRMRQ
AN6557.1  	MTINPTYLAQRTRSS1ANWSDAKKRVLRSYRDWLRA0SPEIQTMYSLNLPVSAIRTKVRQ
          	*.*.**  *  * .* ***:***:**:  ** *:*: :**:*:**.:  *:*.***::**

MG06310.1 	EFERHRYVNKLSVVDVLLFQSGADYQ0ETMNFWRQTTHIMSYFPEENFRGEKRLPKSFME
NCU00418.1	EFERNRFVNKLPVVDVLLTKGHADYQ0ETMNFWRQTTHMMSYFNEESFRGAKRLPSSFID
FG06936.1 	EFERQRFVNKLSVVDVLLFKSHAEYQ0ETMNFWKQTNHIMAYFKEENFRGDKRLPSSFMT
AN6557.1  	EFEKHRYVSQLQVVDVLLYQSHSEFQ0ETLNYWKQLSHVMKYFRPEEDPG-ARLPPNFIS
          	***::*:*.:* ****** :. :::* **:*:*:* .*:* **  *.  *  *** .*: 

MG06310.1 	GFLE0GRN-------
NCU00418.1	GFLQ~VSLYMQLFCL
FG06936.1 	GFLE0GRN-------
AN6557.1  	GFLE~GRN-------
          	***:
```
